# Cine-ASL: a new arterial spin labeling method for myocardial perfusion mapping in mice using a Cine-FLASH labeling and readout module

**DOI:** 10.1186/1532-429X-14-S1-O74

**Published:** 2012-02-01

**Authors:** Thomas Troalen, Thibaut Capron, Monique Bernard, Patrick Cozzone, Frank Kober

**Affiliations:** 1 Centre de Résonance Magnétique Biologique et Médicale, UMR CNRS n°6612, Faculté de Médecine, Aix-Marseille Université, Marseille, France

## Summary

This study presents a novel arterial spin labeling method to assess myocardial perfusion in small rodents. An ECG-gated pseudo-continuous labeling approach is combined with simultaneous readout over the cardiac cycle using Cine-FLASH. This method allows shorter acquisition times than the previously used Look-Locker FAIR gradient-echo technique while preserving spatial resolution and robustness with respect to cardiac motion. Perfusion measurements were carried out on five mice with both techniques under different anesthetic conditions. Perfusion maps and values show good accordance between both experiments.

## Background

Arterial spin labeling (ASL) has been developed and used to quantitatively map rodent myocardial blood flow (MBF) for more than a decade. There is growing interest in improving sensitivity and measurement strategy, because accurate and motion-robust acquisitions are still time-consuming with typical measurement times of about 20 minutes. Here, we propose to combine continuous Cine-MRI gradient echo readout with a pseudo-continuous arterial labeling approach, which leads to a perfusion-dependent stationary regime.

## Methods

**Figure 1:**
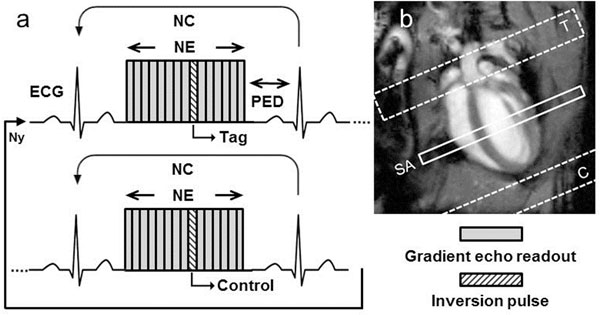
ECG-gated Cine-FLASH ASL (a). NE: number of echoes, NC: number of cine block repetitions, PED: Pre-ECG delay, Ny: number of phase encoding steps. Resolution = 195×391 µm^2^, TE = 1.64 ms, TR = 8ms, slice thickness 1.5 mm, acquisition time 8 min at 500 bpm. Labeling plane disposition on a four chambers view (b). T: Tag, C: Control, SA: Short-Axis imaging slice.

Female C57BL/6 mice (weight = 24-30g, n=5) were anesthetized with 1.5% isoflurane added to 1.2l/min of pure O2. MBF was assessed on a Bruker Biospec 4.7T MR system using an ECG-gated Cine-FLASH sequence repeated over several cardiac cycles in order to reach the steady-state of a FLASH experiment under the influence of perfusion. As shown in figure 1, one echo in each cine block was substituted by an inversion pulse, labeling the arterial blood in a slice covering the aortic arch and atriums at a precise timing within the cardiac cycle. A control scan was achieved by placing the inversion slab in symmetry to the short-axis imaging plane, compensating for magnetization transfer effects. Interleaved tag and control acquisitions are performed for each k-space line with a total acquisition time of 8 minutes. For comparison, a Look-Locker-FAIR gradient echo ASL (LLFAIRGE) measurement was performed (acquisition time 25 minutes). In two mice, both experiments were performed under two different isoflurane concentrations 1.5% and 2%, the latter inducing strong vasodilation. For both methods, perfusion maps were calculated, and MBF was measured in a region of interest placed into the anterior wall of the myocardium.

## Results

**Figure 2:**
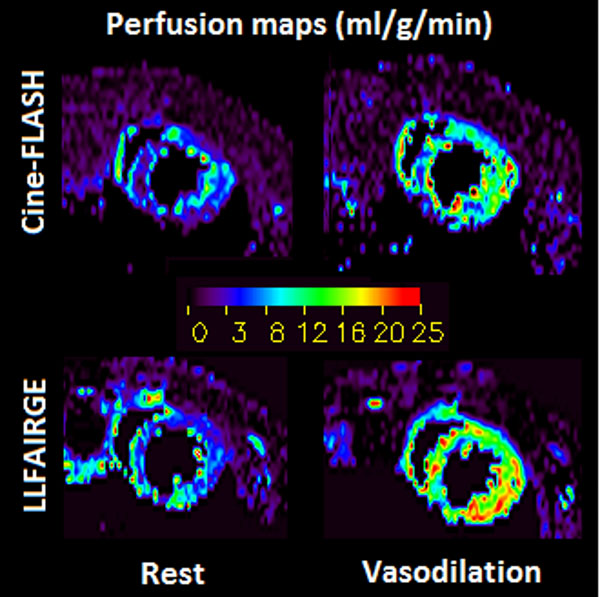
Perfusion maps obtained from a mouse at rest (left column) and during vasodilation (right column). The upper row show results obtained with the new Cine-FLASH ASL approach while the lower row presents perfusion assessed with LLFAIRGE sequence. With both methods, a significant increase in myocardial blood flow can be seen, with lower perfusion values for the Cine-FLASH ASL experiment.

The results showed good concordance between myocardial perfusion assessed with the new Cine-FLASH ASL technique (8.9 ± 2.5 ml g-1 min-1) and LLFAIRGE (9.8 ± 1.8 ml g-1 min-1), although the new technique had a tendency to lower values. Globally, all mice evaluated here showed relatively high perfusion values compared with previous studies. Under vasodilatation conditions induced by high isoflurane concentration, a significant increase in MBF was consistently observed with both techniques (figure 2). Theoretical calculations setup for this Cine-ASL sequence show that during each acquisition block (control and tag), a steady state magnetization is rapidly reached at which the magnetization difference between tag and control scans depends explicitly on perfusion while it is independent of uncontrolled dynamic details of the experiment such as arterial input function and heart rate variations.

## Conclusions

We present a non-invasive and accurate MRI method to quantify MBF in vivo in mice using a new labeling and readout approach. The steady-state Cine-FLASH readout module allows for averaging of the steady perfusion-dependent magnetization difference over several cardiac cycles and can be used to improve sensitivity.

## Funding

CNRS, UMR 6612.

Agence Nationale de la Recherche -08-BLANC-0058-01.

Ph.D. Grant Siemens France (TT).

